# Twist-2 Controls Myeloid Lineage Development and Function

**DOI:** 10.1371/journal.pbio.0060316

**Published:** 2008-12-16

**Authors:** Andrew B Sharabi, Melissa Aldrich, Drazen Sosic, Eric N Olson, Alan D Friedman, Sung-Hyung Lee, Si-Yi Chen

**Affiliations:** 1 Department of Immunology, Center for Cell and Gene Therapy, Baylor College of Medicine, Houston, Texas, United States of America; 2 Department of Molecular Biology, University of Texas Southwestern Medical Center, Dallas, Texas, United States of America; 3 Division of Pediatric Oncology, Johns Hopkins University, Baltimore, Maryland, United States of America; 4 Department of Molecular Microbiology and Immunology, Norris Comprehensive Cancer Center, Keck School of Medicine, University of Southern California, Los Angeles, California, United States of America; University of Wisconsin-Madison, United States of America

## Abstract

Basic helix-loop-helix (bHLH) transcription factors play critical roles in lymphoid and erythroid development; however, little is known about their role in myeloid lineage development. In this study, we identify the bHLH transcription factor Twist-2 as a key negative regulator of myeloid lineage development, as manifested by marked increases in mature myeloid populations of macrophages, neutrophils, and basophils in Twist-2–deficient mice. Mechanistic studies demonstrate that Twist-2 inhibits the proliferation as well as differentiation of granulocyte macrophage progenitors (GMP) by interacting with and inhibiting the transcription factors Runx1 and C/EBPα. Moreover, Twist-2 was found to have a contrasting effect on cytokine production: inhibiting the production of proinflammatory cytokines such as interleukin-12 (IL-12) and interferon-γ (IFNγ) while promoting the regulatory cytokine IL-10 by myeloid cells. The data from further analyses suggest that Twist-2 activates the transcription factor c-Maf, leading to IL-10 expression. In addition, Twist-2 was found to be essential for endotoxin tolerance. Thus, this study reveals the critical role of Twist-2 in regulating the development of myeloid lineages, as well as the function and inflammatory responses of mature myeloid cells.

## Author Summary

Hematopoiesis is coordinated by transcription factors that regulate proliferation, differentiation, and cell fate determinations. Myelopoiesis refers to the development of all white blood cells, excluding lymphocytes (B and T cells); however, the molecular regulation of this developmental process is still incompletely understood. In this study using mice that lack expression of Twist-2, we establish a novel role for this basic helix-loop-helix transcription factor as regulator of myeloid progenitors and fully differentiated myeloid cells. Specifically, Twist-2 acts to inhibit proliferation as well as differentiation of progenitors that give rise to macrophages, neutrophils, and basophils by inhibiting the important transcription factors Runx1 and C/EBPα. In mature myeloid cells, Twist-2 negatively regulates the production of proinflammatory cytokines while positively promoting the production of regulatory cytokine IL-10 by these cells. These findings provide significant insight into regulation of myeloid lineage development and function.

## Abstract 3

The transcription factor Twist-2 is a new regulator that inhibits the proliferation and differentiation of granulocyte macrophage progenitors. Twist-2 also inhibits proinflammatory cytokine production, while stimulating IL-10 by myeloid cells.

## Introduction

Hematopoietic cell development and function must be tightly regulated to maintain homeostasis. Cell fates are established by master transcription factors that orchestrate determination and differentiation. Disruption of this regulation can lead to lethal consequences for the host in the form of myelodysplasias or leukemia. Development of the terminally differentiated myeloid lineages follows a hierarchy starting with the hematopoietic stem cell (HSC) [1–3], which gives rise to a series of rapidly dividing committed progenitors [4,5], namely the common myeloid progenitor (CMP) and granulocyte macrophage progenitor (GMP). The identification of specific surface markers has allowed for prospective isolation of these populations and has facilitated investigation of the transcriptional regulation that occurs during myelopoiesis [6–8]. Transcription factors, including PU.1 and C/EBPα, play critical roles in development of myeloid lineages because in the absence of these factors, specific populations fail to develop or are severely altered. PU.1 plays a broad role in determination of both myeloid and lymphoid lineages, as mice deficient in PU.1 fail to develop B cells, T cells, granulocytes, or macrophages [9]. C/EBPα is necessary for proper granulocyte colony-stimulating factor receptor (G-CSFR) promoter transactivation as well as additional downstream activities, and is hence required for formation of the GMP and myeloid lineage commitment [10–14]. Although many transcription factors have been identified that play critical roles in promoting myeloid lineage development, factors that naturally function to inhibit or negatively regulate myeloid lineage development are largely unknown and require further characterization. These inhibitory factors may play equally important roles in regulating hematopoiesis by preventing excessive myeloid lineage development or myeloproliferative disease. Furthermore, aberrant expression of inhibitory factors, as exemplified by the Runx1-ETO fusion protein, which functions as a dominant-negative regulator of the transcription factor Runx1 and an inhibitor of the C/EBPα promoter [15,16], may play a direct role in leukemogenesis by blocking normal differentiation and creating an enlarged pool of progenitors that are prone to malignant transformation.

Importantly, many basic helix-loop-helix (bHLH) transcription factors, including the E2A family, stem cell leukemia factor (SCL/Tal1), Lyl-1, and the helix-loop-helix (HLH) Id family, are known to be important regulators of hematopoiesis [17–21]. bHLH factors form heterodimers or homodimers that can bind E-box DNA consensus sites comprised of the sequence 5′-CANNTG-3′. SCL is a bHLH factor that is required for definitive hematopoiesis [18,19]. SCL knockout (KO) mice are embryonic lethal due to a failure in primitive yolk sac hematopoiesis, and conditional KO mice have defective erythropoiesis and megakaryopoiesis, but myeloid lineages are largely unaffected [22]. Lyl-1 is a bHLH factor, closely related to SCL, which is important for proper B cell differentiation and regulates the reconstitution ability of HSCs [20]. The E2A bHLH family is another group that plays an essential role in development of B cell lineages, without which B cell development is arrested at the pre-pro B cell stage [23,24]. A unique set of HLH proteins is the Id family, which lacks the basic region required for DNA binding and function as dominant-negative regulators of other HLH factors. Id-2 and Id-3 cross-repress E2A-mediated B cell development to skew lymphoid dendritic cell (DC) or natural killer (NK) cell lineages [25–27].

Nevertheless, whereas many HLH factors have been found to be essential for proper lymphoid or erythroid development, the role of HLH factors in myeloid lineage development is less understood. Furthermore, in general, very few intrinsic negative regulators of myeloid lineage development have been identified. Instead, much of the inhibitory regulation that is reported is the result of competition or cross-inhibition between transcription factors such as PU.1 and C/EBPα, or between PU.1 and Gata-1 [28]. Thus, we hypothesized that there are unknown bHLH transcription factors that regulate myeloid lineage development. In this study, we identify the bHLH factor Twist-2 as a critical factor that regulates the proliferation and differentiation of myeloid lineage progenitors, as well as the function and inflammatory responses of mature myeloid cells.

## Results

### Preferential Expression of Twist-2 in Myeloid Progenitors

**Figure 1:**
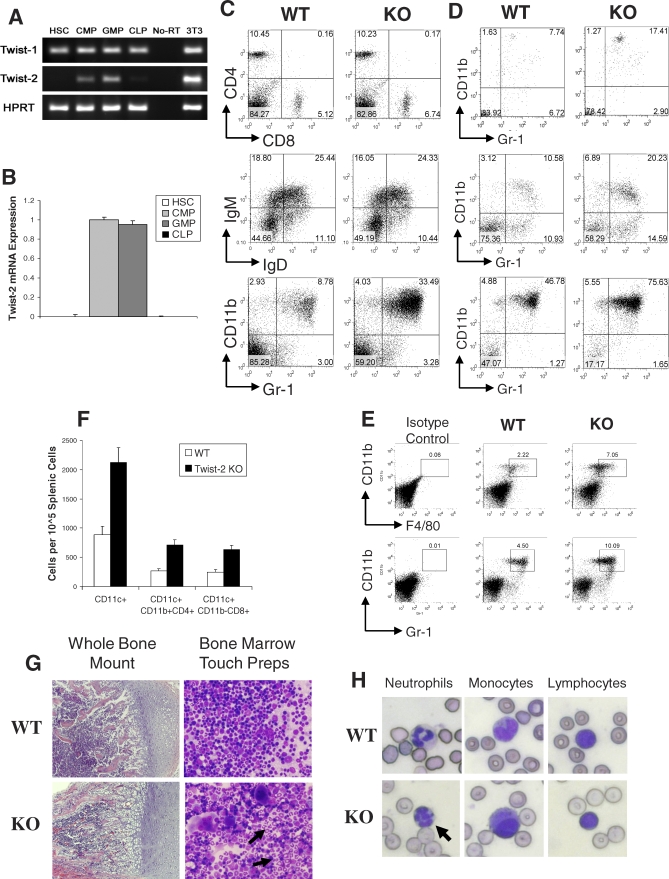
Twist-2–Deficient Mice Have Significant Increases in Multiple Myeloid Cells in Multiple Organs (A and B) Semiquantitative (A) and quantitative (B) RT-PCR of sorted hematopoietic progenitor populations showing constitutive expression of Twist-2 in GMP and CMP populations. Data are normalized to 18s rRNA internal controls and representative of two independent experiments. Error bars indicate the standard deviation (SD). (C) Splenocytes of Twist-2 KO and WT littermates were analyzed by flow cytometry. Results are representative of eight independent pairs of mice analyzed. Splenic CD4^+^ and CD8^+^ T cells (upper panel), IgM^+^ and IgD^+^ B cells (middle panel), and CD11b^+^ and Gr-1^+^ granulocytes and macrophages (lower panel) are shown. (D) Flow cytometry showing expansion of CD11b^+^Gr-1^+^ myeloid cells in peripheral blood (upper panel), liver (middle panel), and BM (lower panel) of Twist-2 KO mice representative of six independent experiments. (E) Flow cytometric analysis of splenocytes showing the expansion of Gr-1^low^CD11b^+^ F4/80^high^ macrophages (upper panel) and F4/80^low^CD11b^+^Gr-1^high^ neutrophils (lower panel) in Twist-2 KO mice representative of three independent experiments. (F) Absolute cell counts of CD11c^+^ cells and two major DC subtypes, CD11c^+^CD11b^+^CD4^+^ and CD11c^+^CD11b^−^CD8^+^ cells, are both increased in Twist-2 KO spleen. Error bars indicate SD. (G) Images of whole bone mounts stained with H&E (left panels). BM touch preps stained with Wright-Giemsa (right panels) show higher numbers of mature myeloid cells in the Twist-2 KO BM. (H) Images of peripheral blood smears with Wright-Giemsa stain showing hypersegmented neutrophils and enlarged monocytoid cells in Twist-2 KO mice. Images are representative of four independent sets of peripheral blood smears. The arrow indicates the hypersegmented nuclei of the neutrophils.

In order to identify bHLH factors that regulate myeloid development, we first used Gene Expression Omnibus (GEO) microarray dataset GSE3722 to analyze the expression of a panel of candidate genes, including known myeloid transcription factors and bHLH factors in CMP and GMP (Figure S1). Subsequent semiquantitative reverse transcription PCR (RT-PCR) analysis of sorted hematopoietic progenitors from wild-type (WT) C57B/6 mice (Figure S1) showed that Twist-2 was preferentially expressed in Lin^−^IL7R^−^Sca-1^−^c-Kit^+^CD34^+^FcγRII/III^high^ GMP and Lin^−^IL7R^−^Sca-1^−^c-Kit^+^CD34^+^FcγRII/III^−^ CMP, but not or weakly expressed in the Lin^−^IL7R^−^Sca-1^+^c-Kit^+^ hematopoietic progenitor compartment containing HSC or Lin^−^IL7R^+^Sca-1^low^c-Kit^low^ common lymphoid progenitors (CLP) (Figure 1A). In contrast to the preferential expression of Twist-2, Twist-1 was expressed in all types of progenitors we examined (Figure 1A). Quantitative RT-PCR assays further confirmed the preferential and constitutive expression of Twist-2 in CMP and GMP populations (Figure 1B).

### Twist-2 KO Mice Have Significant Systemic Increases in Multiple Myeloid Lineages

The Twist family of bHLH transcription factors, including Twist-1 and Twist-2, are key regulators of mesodermal differentiation [33] and also play a role in the epithelial to mesenchymal transition involved in cancer metastasis [34]. Twist-2 is known to inhibit the terminal differentiation of a variety of mesodermally derived cell types including myocytes, osteoblasts, and adipocytes [29–32]. Although many of the known HLH factors are direct dimerization partners with Twist-2, there are no reports, to our knowledge, investigating the role of Twist-2 in hematopoiesis.

To investigate the possible role of Twist-2 in hematopoiesis, we enumerated mature myeloid cells and other types of immune cells from lymphoid organs in age- and sex-matched Twist-2 knockout (KO) (^−/−^) mice and wild-type (^+/+^) littermates. Twist-2 KO mice develop a severe inflammatory syndrome, and the majority of mice die within 2 wk after birth [35]. This phenotype is associated with elevated serum levels of the cytokines interleukin-1 (IL-1) and tumor necrosis factor-α (TNFα) due to derepression of necrosis factor κB (NF-κB) activity [35,36]. Upon analyzing Twist-2 KO mice, we observed similar percentages of T cells and B cells in the spleens as compared to WT littermates (Figure 1C). However, we found a significant increase in CD11b^+^Gr-1^+^ myeloid cells in the spleens of Twist-2 KO mice (Figure 1C, lower panel). In order to determine whether this myeloid population was systemically increased, we analyzed the liver, blood, and bone marrow (BM), and found increases in CD11b^+^Gr-1^+^ populations in each of these tissues and organs of Twist-2 KO mice (Figure 1D). Using the specific macrophage marker F4/80, we identified this increased population to include both CD11b^+^Gr-1^low^F4/80^+^ macrophages and CD11b^+^Gr-1^high^F4/80^−^ neutrophils (Figure 1E). In addition, we observed an increase in CD11c^+^ myeloid DCs in spleens of Twist-2 KO mice, with equal contributions to two major DC subtypes, namely CD11c^+^CD11b^+^CD4^+^ and CD11c^+^CD11b^−^CD8^+^ DCs (Figure 1F).

Multiple different cell types of the myeloid lineage stain positive with CD11b and Gr-1 antibodies, and so in order to distinguish these myeloid cell subpopulations, we performed automated complete blood counts (CBC) with differentials on peripheral blood obtained from a series of eight Twist-2 KO mice and WT littermates. Twist-2 KO mice have increased percentages and absolute cell counts of multiple myeloid lineages, including a 2–3-fold increase in macrophages and neutrophils as well as an 18-fold increase in absolute basophil count (Table 1). There was no apparent change in absolute lymphocyte, red blood cell, or platelet counts in peripheral blood, suggesting that other lineages, including megakaryocyte and erythroid lineages, were not significantly affected. Histology of whole bone mounts demonstrated a largely intact architecture of the trabecular bone in Twist-2 KO mice (Figure 1G, left panel). Histological analysis of Twist-2 KO BM revealed increases in myeloid cells in multiple stages of maturation (Figure 1G, right panel and arrows), which is consistent with the CD11b^+^Gr-1^high^ phenotype of the expanded myeloid populations (Figure 1C–1F). Interestingly, we observed that hypersegmented neutrophils and atypical monocytes were present in peripheral blood smears of Twist-2 KO mice (Figure 1H), which are pathologic findings that can be observed in the setting of myelodysplastic and myeloproliferative diseases [37]. Furthermore, the dramatic increase in basophils is suggestive of a myeloproliferative disease because basophilia is mainly observed in the setting of myeloproliferative disease and cases of chronic myeloid leukemia [38,39].

**Table 1:**
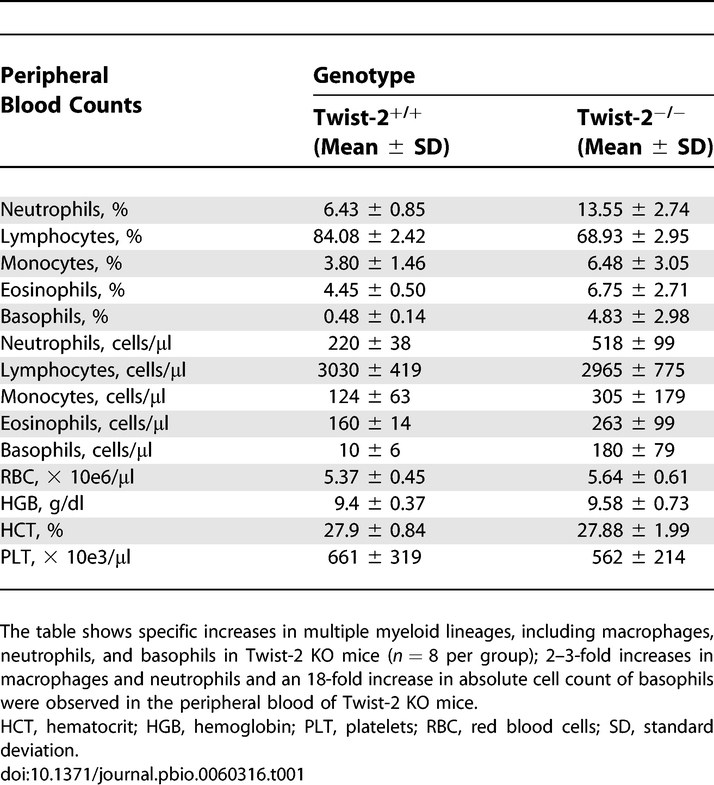
Complete Blood Counts with Differentials on Peripheral Blood

### Increases in Myeloid Cells Due to an Intrinsic Feature of Hematopoietic Progenitors Deficient in Twist-2

**Figure 2:**
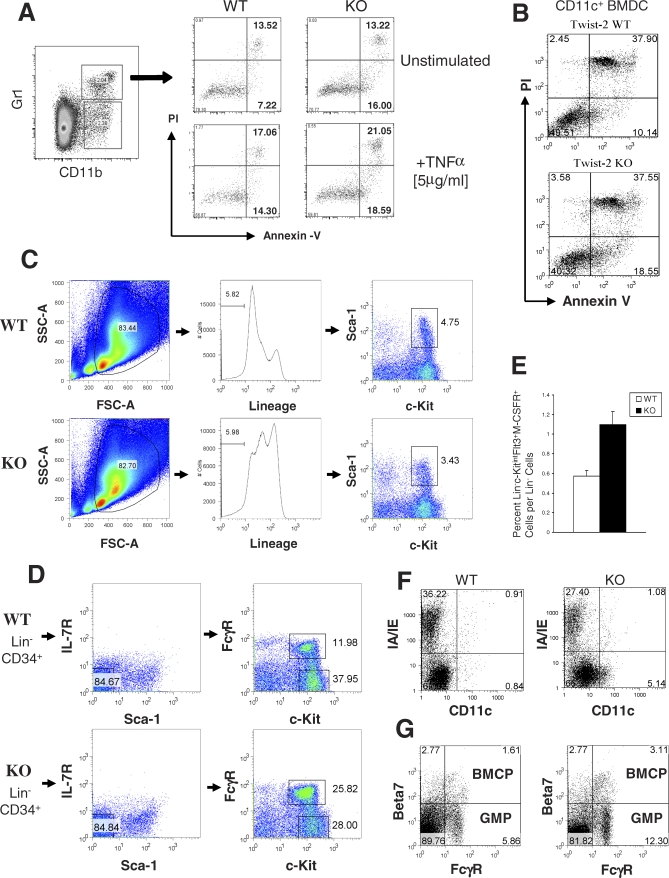
Increased Myeloid Progenitors in Twist-2–Deficient Mice (A) Flow cytometry of splenocytes cultured in complete medium and stimulated with TNFα (5 μg/ml) for 24 h. Cells were stained with CD11b, Gr-1, and Annexin-V antibodies and PI to determine relative apoptotic and necrotic populations from one of the representative repeated experiments. (B) Flow cytometry of BMDC derived from Twist-2 KO mice or WT littermates at day 10 of culture from one of the representative repeated experiments. Cells were stained with CD11c and Annexin-V antibodies and PI. (C) Flow cytometric analysis showing a subtle decrease in Lin^−^c-Kit^+^Sca1^+^ HSC compartment in Twist-2 KO mice. Results are representative of four independent experiments. (D) Flow cytometric analysis showing a significant increase in Lin^−^IL7R^−^Sca1^−^c-Kit^+^CD34^+^FcγR^+^ GMP population in BM of Twist-2 KO mice representative of six independent experiments. (E) Flow cytometric analysis showing a significant increase in Lin^−^c-Kit^int^Flt3^+^M-CSFR^+^ DC precursors in the BM of Twist-2 KO mice. Representative of two independent experiments. Error bars indicate SD. (F and G) Twist-2 KO mice have increased IA/IE^−^CD11c^+^ DC precursors ([F]) in the peripheral blood and increased Lin^−^IL7R^−^c-Kit^+^FcγR^+^Beta7^high^ BMCP as well as Lin^−^IL7R^−^c-Kit^+^FcγR^+^Beta7^−/lo^ GMP in Twist-2 KO spleen (G). Results are representative of three independent experiments.

To mechanistically investigate the increases in myeloid cells observed in Twist-2 KO mice, we first tested whether a decrease in apoptosis or cell turnover of these myeloid cells in the periphery results in their accumulation. Splenocytes from Twist-2 KO and WT littermates were cultured in complete media and stimulated with proapoptotic cytokines. Cells were then analyzed by flow cytometry with staining for Annexin-V and propidium iodide (PI). Twist-2 KO CD11b^+^Gr-1^+^ cells did not show any increased resistance to cell death. In fact, there was a slightly increased susceptibility to apoptosis as indicated by the increased percentage of Annexin-V^+^PI^−^ Twist-2 KO cells (Figure 2A). In addition, we did not observe any apparent difference in the percentage of CD11c^+^Annexin-V^+^PI^+^ late apoptotic or necrotic DCs derived from Twist-2 KO and WT BMs (Figure 2B). Thus, it is unlikely that the increased populations of myeloid cells we observed in Twist-2 KO mice are due to decreased cell turnover or resistance to apoptosis in these populations. Another possibility is that a change in cell trafficking may result in relative increases of these myeloid cells in specific organs or tissues at the expense of others. However, this possibility was largely excluded due to the observed increases in multiple tissues and organs of Twist-2 KO mice, including the BM. The possibility of increased myeloid cells as a result of infection was also unlikely, due to the lack of evidence of infection in these mice and to their housing in a pathogen-free barrier facility. Next, we examined the possibility that altered or dysregulated hematopoiesis in Twist-2 KO mice results in increased production or myeloproliferation.

### Increases in Myeloid Lineage Progenitors in Twist-2–Deficient Mice

To investigate the intrinsic mechanism for the observed increases in myeloid cells in Twist-2 KO mice, we used flow cytometric analysis to examine the HSC and progenitor populations. We observed a subtle decrease in the Lin^−^c-Kit^+^Sca-1^+^ HSC compartment and percentage of Lin^−^IL7R^−^Sca-1^−^c-Kit^+^CD34^+^FcγRII/III^−^ CMP in Twist-2 KO BM (Figure 2C and 2D). In contrast, we found a significant expansion of the Lin^−^IL7R^−^Sca-1^−^c-Kit^+^CD34^+^FcγRII/III^high^ GMP population in the BM and spleens of Twist-2 KO mice (Figure 2D and 2G).

Downstream progenitors and immediate myeloid precursors could also contribute to myeloproliferation in Twist-2 KO mice. Recently, there has been significant progress in identifying specific surface markers of these precursor populations and delineating their hierarchical program of differentiation [7,40–42]. Mouse DC precursors in the BM have been defined as being Lin^−^c-Kit^int^Flt3^+^MCSFR^+^ [43,44], and mouse DC precursors in the peripheral blood have been also immunophenotypically defined as being CD11c^+^IA-E^−^ [41]. We stained for these cells in Twist-2 KO mice and WT littermates, and found significant increases in Lin^−^c-Kit^int^Flt3^+^MCSFR^+^ and CD11c^+^IA-E^−^ DC precursors in the BM and peripheral blood of Twist-2 KO mice, respectively (Figure 2E and 2F). Recently, a basophil mast cell progenitor (BMCP) has been identified in the mouse spleen that is characterized as being Lin^−^c-Kit^+^FcγR^+^Beta7^+^ [42]. The addition of the Beta7^+^ marker appears to fractionate BMCP from Lin^−^c-Kit^+^FcγR^+^Beta7^−^ GMP. Importantly, we observed an increase in BMCP in the spleens of Twist-2 KO mice (Figure 2G). Taken together, these data indicate that Twist-2 plays a role in negatively regulating differentiation and development of myeloid lineages in vivo.

### Twist-2–Deficient Hematopoietic Progenitors Are Skewed toward Myeloid Differentiation In Vitro and In Vivo

**Figure 3:**
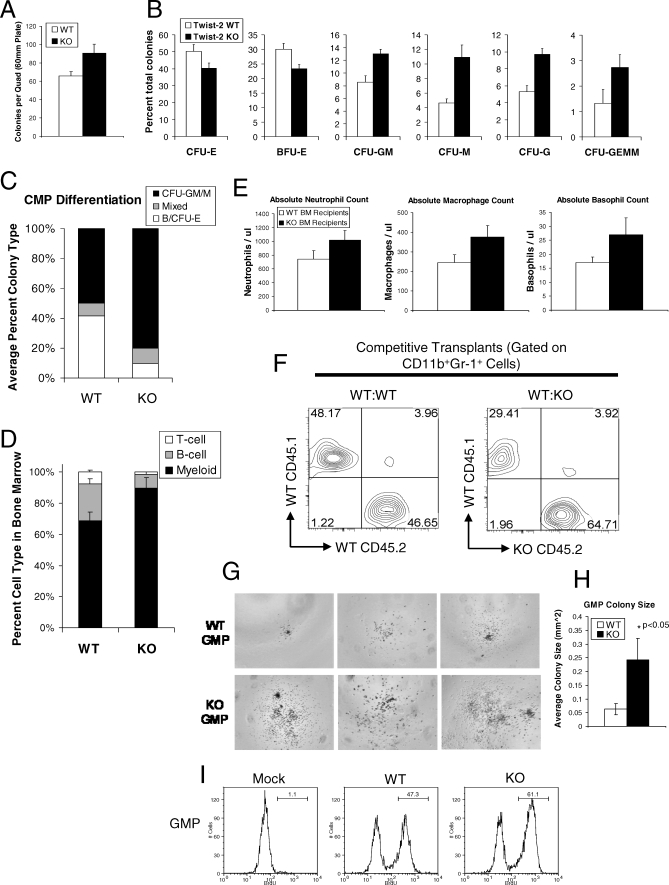
Twist-2 Regulates the Myeloid Differentiation and Proliferation of Hematopoietic Progenitors in an Intrinsic and Cell Autonomous Manner (A and B) Twist-2 KO BM cells are hypersensitive to GM-CSF–induced colony formation and skewed toward the differentiation of myeloid colonies. Cells cultured in MethoCult M3001 containing GM-CSF only (A), and MethoCult GF M3434 containing a growth factor and cytokine cocktail (B) in vitro are shown. Colonies were counted on day 12 of culture (*n* = 5 plates per group), and results are representative of three independent experiments. (C) Single-cell Lin^−^IL7R^−^Sca-1^−^c-Kit^+^CD34^+^FcγRII/III^−^ CMPs from Twist-2 KO mice or WT littermate BM were sorted into individual wells of a 96-well plate containing MethoCult and cultured for 10 d. Resultant colonies were scored by their respective type (BFU-E/CFU-E, mixed GEMM, and CFU-GM/CFU-M). Data are representative of three independent experiments. (D and E) Transplantation of Twist-2 KO BM results in increased myeloid cell counts in irradiated syngeneic hosts. Whole BM from Twist-2 KO mice or WT littermates was injected retro-orbitally into lethally irradiated syngenic mice (2 × 10^6^ cells/mouse, *n* = 6 per group). After 4 wk, BM was analyzed and relative percentages of CD4^+^ and CD8^+^ T cells, B220^+^Gr-1^−^CD11b^−^ B cells, and B220^−^Gr-1^+^CD11b^+^ myeloid cells were calculated (D). Peripheral blood was analyzed in a separate cohort at 8 wk by automated CBC with differential (E). Data are representative of two independent experiments. (F) For competitive transplants, whole BM from CD45.2 Twist-2 KO mice or CD45.2 WT littermates was mixed 1:1 with WT CD45.1 BM and injected retro-orbitally into lethally irradiated CD45.1 congenic mice (400 × 10^3^ cells/mouse, *n* = 5 per group). After 4 wk, BM was analyzed, and relative percentages of Gr-1^+^CD11b^+^ myeloid cells expressing CD45.1^+^ and CD45.2^+^ were analyzed. Results are representative of two independent experiments. (G and H) Increased size and number of myeloid colonies of Twist-2 KO GMP in vitro. Single-cell Lin^−^IL7R^−^Sca-1^−^c-Kit^+^CD34^+^FcγRII/III^high^ GMPs were sorted directly into individual wells of 96-well plates containing MethoCult (GF M3434) and cultured for 5 d. Images are representative of three independent experiments (G). Colonies were imaged and digitally analyzed (*n* = 20 colonies per group) (H). Error bars indicate SD. (I) Twist-2 KO GMPs are hyperproliferative in vivo. Twist-2 KO and WT mice were injected intraperitoneally with 170 μg/kg BrdU or PBS. After 2 h, mice were sacrificed and BM was stained for GMP surface markers and BrdU. Proliferating BrdU^+^ populations in the gated Lin^−^IL7R^−^Sca-1^−^c-Kit^+^CD34^+^FcγRII/III^high^ GMPs are shown from one of three repeated experiments.

To further investigate whether Twist-2 functions as a suppressor of myeloid cell lineage differentiation, we first used methylcellulose colony formation assays to analyze the relative ability of the BM from WT and Twist-2 KO littermates to differentiate into hematopoietic colonies in the presence of granulocyte-macrophage colony-stimulating factor (GM-CSF) in vitro. Figure 3A shows that BM from Twist-2 KO mice produced a significantly increased number of total colonies, suggesting a hypersensitivity to GM-CSF–induced differentiation or proliferation (Figure 3A). Next, we analyzed the types of colonies produced upon hematopoietic differentiation in MethoCult GF M3434 (StemCell Technologies), which supports differentiation of multiple hematopoietic lineages in vitro. Under these conditions, we observed a significant increase in the relative percentage of myeloid colonies (colony-forming unit granulocyte macrophage [CFU-GM], CFU macrophage [CFU-M], and CFU granulocyte [CFU-G]) as identified by their characteristic morphologies (Figure 3B). Interestingly, when analyzing the resulting colonies by flow cytometry, we observed an increase in Lin^−^Sca-1^−^c-Kit^+^ cells, suggesting an increased maintenance or proliferation of myeloerythroid progenitors (unpublished data). In addition, we observed increased percentages of both CFU-GM and CFU-M differentiated in vitro from mouse embryonic stem cells in which Twist-2 was specifically silenced by short interfering RNA (siRNA) (unpublished data).

To examine whether Twist-2 regulates myeloid skewing at the single-cell level, we sorted the upstream CMPs, which have both myeloid and erythroid potential, into individual wells containing MethoCult GF M3434 and cultured the cells for 10 d. Resultant colonies were scored for their respective type (BFU-E/CFU-E, mixed GEMM, and CFU-GM/CFU-M). In agreement with in vitro differentiation assays using whole BM cells (Figure 3A and 3B), Twist-2 KO CMP cells were clearly skewed towards differentiating into myeloid colonies (Figure 3C). Next, we performed competitive and noncompetitive BM transplantation assays to assess the intrinsic effects of Twist-2 during hematopoiesis. Lethally irradiated syngeneic WT mice receiving Twist-2 KO BM had a significantly expanded myeloid compartment in the BM at 4 wk (Figure 3D). Absolute cell counts of multiple myeloid lineages in the peripheral blood were increased at 8 wk after transplantation as compared to control mice receiving WT BM (Figure 3E). Since it is possible that the downstream effects of Twist-2 on cytokine production could alter hematopoiesis, we performed competitive BM transplantation assays using congenic markers to assess the independent effects of Twist-2 on hematopoiesis in vivo. We admixed WT CD45.1 BM 1:1 with either WT CD45.2 or Twist-2 KO CD45.2 BM, and transplanted the cells into lethally irradiated CD45.1 congenic mice. Upon analyzing the mice at 4 wk, we observed a relatively equal contribution to the CD11b^+^Gr-1^+^ myeloid compartment in mice that received the WT:WT BM (Figure 3F). However, in mice that received the WT:KO admixed BM, an enhanced fraction of the CD11b^+^Gr-1^+^ myeloid cells was derived from the Twist-2 KO BM, indicating that the Twist-2 KO cells have a competitive advantage over WT cells, given the same in vivo microenvironment (Figure 3F, right panel). Collectively, these data demonstrate the skewed myeloid lineage differentiation of Twist-2–deficient progenitor cells in vitro and in vivo, and indicate that Twist-2 is likely an intrinsic inhibitor of myeloid differentiation.

### Hyperproliferation of Twist-2–Deficient GMP Cells

To examine whether Twist-2 also plays a role in regulating the proliferation of myeloid progenitor cells, we first sorted single cells of Lin^−^IL7R^−^Sca-1^−^c-Kit^+^CD34^+^FcγRII/III^high^ GMP from the BM of Twist-2 KO and WT littermates into individual wells of 96-well plates and analyzed their differentiation and proliferation in MethoCult GF M3434 (StemCell Technologies). It was apparent that the individual colony sizes derived from the Twist-2 KO GMP cells were significantly larger than those from WT GMP cells (Figure 3G). Individual colonies from Twist-2 GMP cells also had increased cell density (unpublished data). In order to quantify this observation, we used an image analysis software package to measure the average size of the colonies. Figure 3H shows that the average size of Twist-2 KO GMP-derived colonies was more than 3.5 times larger than that of WT GMP-derived colonies, indicating the enhanced proliferation and differentiation of Twist-2 KO GMP cells in vitro. To directly examine whether Twist-2 controls GMP proliferation, we performed bromodeoxyuridine (BrdU) labeling assays to monitor the proliferation of GMPs in vivo. Twist-2 KO or WT mice were injected with BrdU, and BM cells were then stained with markers for GMP cells, followed by intracellular staining for BrdU incorporation into newly synthesized DNA. Figure 3I shows that GMP cells from Twist-2 KO mice had increased BrdU incorporation, indicating increased proliferation and cell cycling of this progenitor population in vivo. Taken together, these data indicate that Twist-2 not only suppresses the myeloid differentiation of hematopoietic progenitors, but also specifically inhibits the proliferation of the GMP population.

### Twist-2 Inhibits Runx1/AML1 and C/EBPα

**Figure 4:**
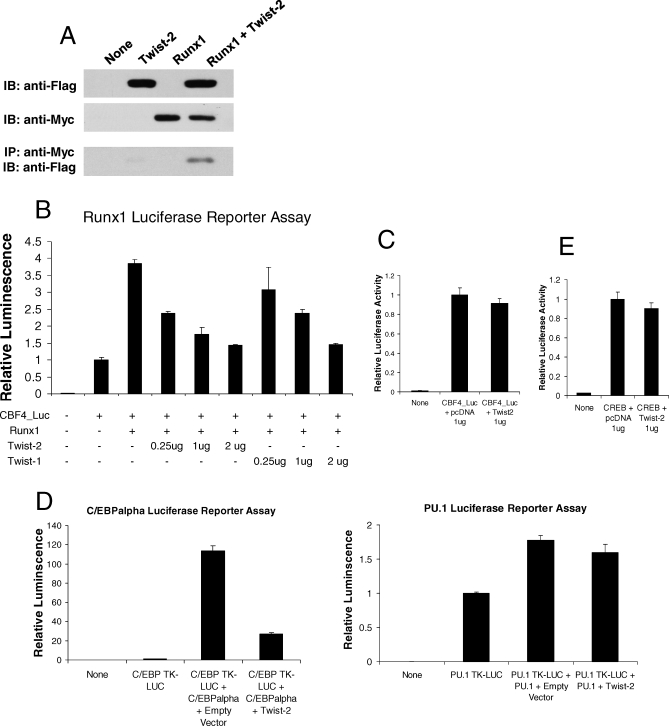
Twist-2 Inhibits the Transcriptional Activity of Runx1 and C/EBPα (A) Immunoassay of COS7 cells transiently transfected with indicated plasmids. After 48 h, cells were lysed in NTN buffer containing 0.5% NP-40 and 0.5% Triton X-100. Cell lysates were analyzed by immunoblotting (IB) with anti-FLAG or anti-myc antibodies (upper two panels). Cell lysates were also immunoprecipitated (IP) with anti-myc antibody followed by immunoblotting with anti-FLAG antibody (lower panel). (B and C) COS7 cells were transiently transfected with a Runx1-responsive luciferase reporter (CBF4_Luc, 1 μg), or Runx1 expression plasmid (Runx1, 1 μg), or indicated amounts of Twist expression constructs. pcDNA3.1 blank vector control was cotransfected so that each well received the same total amount of DNA (4 μg). After 24 h, luciferase activity in cell extracts was quantified via luciferase assay (B). Runx1 responsive luciferase reporter cotransfected with empty vector control or Twist-2 expression vector only (C). Data are presented as mean ± SD and are representative of three independent experiments. (D and E) Luciferase reporter assays were performed on cells transfected with indicated plasmids (1 μg each). Twist-2 vector cotransfection inhibited the activity of a C/EBPα-dependent luciferase reporter in COS7 cells ([D] left panel), but not a PU.1-dependent luciferase reporter ([D] right panel). A CREB-responsive luciferase reporter from the PEPCK promoter cotransfected with empty vector control or Twist-2 expression vector only (E). Data are presented as mean ± SD, and the results are representative of three independent experiments.

Twist-2 forms homodimers or heterodimers with ubiquitously expressed members of the E2A bHLH family [31,45], and can bind E-box consensus sites present in target gene promoters or can directly bind transcriptions factors such as MEF2, Runx2, and NF-κB via its C-terminal domain [31,35,46]. The Twist family has been previously shown to bind the runt homology domain of the Runx2 family member and inhibit its transcriptional activity [46,47]. Given that Runx1 is a known regulator of hematopoiesis, this prompted us to examine whether Runx1 transcriptional activity may be regulated by Twist-2. In order to address this possibility, COS7 cells were transfected with constructs encoding FLAG-tagged Twist-2 and Myc-tagged Runx1. Upon immunoprecipitation of Runx1, we detected Flag-tagged Twist-2 by coimmunoprecipitation (Figure 4A). To investigate the functionality of this interaction, COS7 cells were transfected with a Runx-dependent luciferase reporter construct containing four core binding factor consensus sites. Interestingly, we observed a basal level of reporter activity in COS7 cells, which could be due to a low-level endogenous expression of Runx family members. Cotransfection of Twist-2 resulted in a significant dose-response inhibition of Runx1 reporter activity that was able to reduce the promoter activity to near basal levels (Figure 4B). Neither Twist-2 vector nor empty vector pcDNA alone inhibited the activity of the Runx1-responsive luciferase reporter (CBF4_Luc) (Figure 4C). In addition, neither Twist-2 vector nor empty vector pcDNA inhibited the activity of an unrelated CREB-responsive luciferase reporter (Figure 4E). These data suggest the specificity of Runx1 inhibition by Twist-2. We also observed a subtle increase in Runx1-specific DNA binding in GMP from Twist-2 KO mice as compared to WT controls using electrophoretic mobility shift assays (EMSA) (Figure S2). Since multiple transcription factors play critical roles in myeloid lineage development, we examined the effect of Twist-2 on the transcription factors PU.1 and C/EBPα, which are required for myeloid lineage development. We found that Twist-2 inhibited the transcriptional activity of C/EBPα, but did not significantly affect the transcriptional activity of PU.1 (Figure 4D). These data suggest that Twist-2 suppresses myeloid lineage differentiation and the proliferation of GMP, possibly via inhibition of Runx1 and C/EBPα.

### Twist-2 Suppresses the Production of Proinflammatory Cytokines and Chemokines, Including IL-12

**Figure 5:**
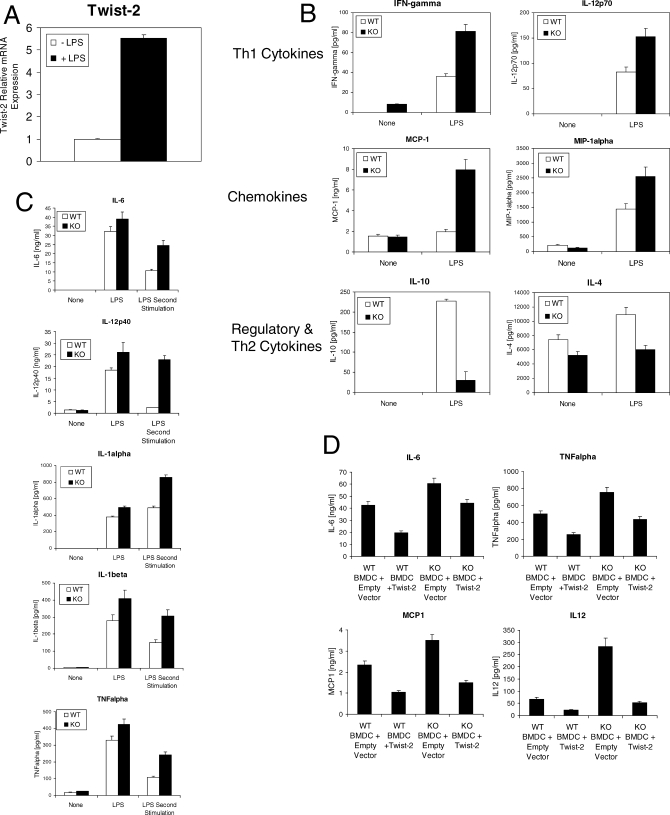
Twist-2 Differentially Regulates the Production of Pro- and Anti-Inflammatory Cytokines by Myeloid Cells (A) Induced expression of Twist-2 mRNA in CD11c^+^ BM DCs after 4 h of stimulation of LPS (100 ng/ml), as demonstrated by quantitative RT-PCR. Data are normalized to 18S rRNA internal controls, with samples loaded in triplicate from repeated experiments. (B) Representative cytokine production by Twist-2 KO and WT DCs after 24 h of stimulation with 100 ng/ml LPS. Data, presented as mean ± SD, are representative of three independent experiments. (C) Endotoxin tolerance assays. DCs were pretreated with 100 ng/ml LPS for 24 h, washed twice in complete medium, and then allowed to rest for 2 h, and then restimulated with 100 ng/ml LPS for an additional 24 h, after which the culture supernatant was collected and assayed. Data are representative of three independent experiments. (D) Representative cytokine production by WT BMDC and Twist-2 KO BMDC transfected with either empty vector control or Twist-2 expression vector. Cells were then stimulated with 100 ng/ml LPS for 24 h. Data presented as mean ± SD are representative of two independent experiments.

By using quantitative RT-PCR analysis, we found that Twist-2 mRNA were expressed in myeloid DCs at low levels, but were rapidly up-regulated in response to stimulation with a Toll-like receptor-4 (TLR4) ligand (LPS) (Figure 5A), which prompted us to investigate whether Twist-2 plays a role in regulation of the function of differentiated myeloid cells. We analyzed the production of inflammatory cytokines by CD11c^+^ splenic myeloid DCs and BM-derived DCs (BMDC) from Twist-2 KO mice using bead-based cytokine arrays and ELISA assays. Figure 5B and 5C show that Twist-2 KO DCs produced elevated levels of the proinflammatory cytokines IL-12, IFNγ, IL-1, TNFα, and IL-6 upon LPS stimulation as compared to WT DC. Production of chemokines, including MCP-1 and MIP-1α, was also significantly increased from Twist-2 KO DCs (Figure 5B). Yet, the surface expression of costimulatory molecules on Twist-2 KO DCs was not significantly altered compared to that on WT DCs (Figure S3). We also set out to examine whether Twist-2 could be involved in mediating endotoxin tolerance, a protection system to prevent harmfully excessive inflammation responses to LPS. DCs from Twist-2 KO and WT littermates were stimulated with LPS for 24 h, then washed and restimulated with LPS to assay for endotoxin tolerance. Figure 5C shows that Twist-2 KO DCs continue to produce high levels of proinflammatory cytokines such as IL-6, TNFα, and IL-12 in response to repeated LPS stimulation. In contrast, WT DCs significantly lost the ability to produce proinflammatory cytokines in response to repeated LPS stimulation. Thus, a biological function of Twist-2 is to maintain endotoxin tolerance to prevent acute excessive inflammatory responses by myeloid cells.

In order to differentiate between the effects of Twist-2 on myeloid cell development versus those on homeostasis in mature progeny, we performed rescue experiments by reexpressing Twist-2 in differentiated KO cells to investigate the reversibility of the proinflammatory cytokine phenotype. BMDCs were generated from WT and Twist-2 KO mice and transfected with a Twist-2 expression vector or empty vector control by Nucleofection (Figure S4). Cells were rested overnight, stimulated with LPS for 24 h, and supernatant was analyzed by ELISA assays. We observed a significant reduction in the production of inflammatory cytokines and chemokines upon rescue of Twist-2 expression in Twist-2 KO BMDC (Figure 5D). We also noticed that overexpression of Twist-2 also suppressed the production of these cytokines and chemokines by WT BMDCs. Thus, these data suggest that the role of Twist-2 in regulating cytokine and chemokine production in differentiated myeloid cells is distinct from its effects on myeloid differentiation.

### Twist-2 Promotes the Production of Anti-Inflammatory Cytokine IL-10

**Figure 6:**
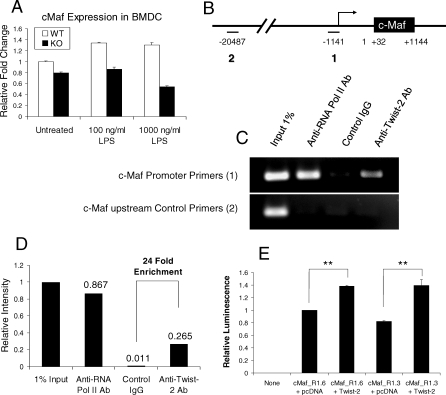
Twist-2 Is an Activator of c-Maf and Essential for Endotoxin Tolerance (A) Quantitative RT-PCR of relative c-Maf mRNA levels in Twist-2 KO and WT BMDCs with or without LPS stimulation from three independent experiments. (B–D) Schematic diagram of c-Maf promoter region and locations of primer pairs: (1) spanning a highly conserved region of the c-Maf promoter containing multiple E-box consensus sites, and (2) spanning a “promoter desert” region of genomic DNA approximately 20 kb upstream of the c-Maf promoter used for the ChIP assay (B). ChIP of 3T3 fibroblasts using an anti–Twist-2 antibody probing for c-Maf promoter DNA pulldown or an anti-RNA Poly (positive) or IgG (negative) controls (C) from one representative of two repeated experiments. Primer sequences available upon request. Densitometric analysis of relative ChIP PCR band intensity (upper panel) was normalized to input (D). (E) Activation of c-Maf promoter by Twist-2. COS7 cells were cotransfected with cMaf promoter-driven luciferase vectors containing approximately 1.6 kb (cMaf_R1.6) or 1.3 kb (cMaf_R1.3) of the rat cMaf promoter region [51] and Twist-2 or empty vector (pcDNA3.1) for 32 h before analyzing for luciferase activity. Double asterisks (**) indicate *p* < 0.01

We unexpectedly found that Twist-2 KO myeloid DCs produced significantly lower levels of the critical anti-inflammatory cytokine IL-10 when compared to WT myeloid DCs (Figure 5B). Further analyses showed that the production of Th2 polarizing IL-4 by Twist-2 KO DCs was also considerably lower (Figure 5B). Twist was previously found to act either as an activator or a repressor of somatic muscle development in *Drosophila* depending upon its dimerization partner [45,48]. To investigate the molecular mechanism by which Twist-2 promotes IL-10 production, we first used an IL-10 promoter-driven luciferase reporter assay. It was observed that Twist-2 did not enhance the IL-10 promoter-derived transcription (unpublished data). Several recent studies have demonstrated that the transcription factor c-Maf is important for the transcription of IL-10 as well as IL-4 [49,50]. Accordingly, we measured the expression of the transcription factor c-Maf in Twist-2 KO and WT DCs. Figure 6A shows that the mRNA levels of c-Maf in Twist-2 KO DCs were significantly lower than those in WT DCs, especially after the stimulation with a TLR agonist, suggesting that Twist-2 is an activator of c-Maf transcription. To further investigate this possibility, we performed chromatin immunoprecipitation (ChIP) assays using 3T3 fibroblasts which express high levels of Twist-2. We found that primers (pair 1) specific for a highly conserved E-box–containing region of the c-Maf promoter (Figure 6B) generated bands from both the RNA-positive control and anti-Twist-2 antibody pulldown, but not control antibody pulldown (Figure 6C). In contrast, negative control primers (pair 2) specific for a region of genomic DNA approx 20 kb upstream of the *c-Maf* gene (Figure 6B and 6C) did not show significant pulldown with any antibody. Densitometry quantified a 24-fold enrichment of specific c-Maf promoter DNA pulldown using Twist-2 antibody as compared to control immunoglobulin G (IgG), suggesting that Twist-2 directly binds the c-Maf promoter (Figure 6D). To further investigate whether Twist-2 is an activator of c-Maf, we cotransfected the expression vectors containing 1.6- or 1.3-kb 5′-flanking region of the *c-maf* gene fused to the luciferase gene [51] with the Twist-2 expression vector or control blank vector into COS cells. Figure 6E shows that Twist-2 activated both *c-maf* promoter constructs. Thus, these data suggest that Twist-2 promotes the production of IL-10 and IL-4, possibly via the activation of c-Maf transcription.

## Discussion

In this study, we found that Twist-2 is constitutively expressed in myeloid progenitors and plays a critical role in the suppression of myeloid lineage differentiation into macrophages, neutrophils, and basophils. We further found that Twist-2 not only inhibits the myeloid differentiation of GMP, but also inhibits the proliferation of GMP population. Moreover, we demonstrated that Twist-2 has inhibitory effects on the activity of Runx1 and C/EBPα, which may contribute to the suppression of GMP differentiation and proliferation by Twist-2. Thus, this study reveals the critical, unrecognized role for Twist-2 in negative regulation of GMP proliferation and myeloid lineage differentiation. In addition, we found that Twist-2 regulates the function and inflammatory responses of differentiated myeloid cells by promoting the production of the important regulatory cytokine IL-10 and inhibiting the production of proinflammatory cytokines and chemokines.

Twist-1 and Twist-2 are highly conserved; however, deficiency in Twist-1 and Twist-2 results in different phenotypes: Twist-1 KO mice are embryonic lethal due to a failure in neural tube closure, whereas Twist-2 KO mice develop a severe inflammatory syndrome and die within 2 wk of birth [35,52]. Twist-1 heterozygous null mice have skeletal and bone defects that mimic patients with Saethre-Chotzen syndrome caused by mutations in human Twist-1 [46,53–55]. Twist-2 is a known developmental regulator of mesodermally derived cell types [29–32]. In this study, we found that Twist-2, but not Twist-1, is selectively expressed in GMP and CMP, and it suppresses GMP differentiation as well as proliferation.

The Runx family comprised of Runx1, Runx2, and Runx3 are master transcription factors that regulate cell-cycle progression and differentiation. Runx1 is essential for definitive hematopoiesis [56–58], and plays an important role in adult hematopoiesis in both lymphoid and myeloid progenitor compartments [59,60]. Runx1 regulates myelopoiesis by coordinating expression of GM-CSF, M-CSFR, myeloperoxidase, and neutrophil elastase [61–64]. In this study, we found that Twist-2 inhibited the function of the transcription factor Runx1, which may contribute to the myeloproliferative disease observed in Twist-2 KO mice. In addition, Runx1 is a target of one of the most common translocations t(8;21) in acute myeloid leukemia (AML), which generates the dominant-negative Runx1-ETO fusion protein to suppress Runx1 activity, leading to the blockade of myeloid lineage differentiation and an accumulated progenitor pool that is prone to malignant transformation [60]. In addition, C/EBPα, a member of the basic region–leucine zipper CCAAT/enhancer-binding protein (C/EBP) transcription factors, is a potent inhibitor of cell cycle in myeloid cells and other types of cells due to binding E2F1. Thus, the suppression of GMP proliferation by Twist-2 may be due to the inhibition of C/EBPα. However, it is possible that Twist-2 interacts with and suppresses additional unrecognized factors. Taken together, our results suggest that Twist-2 suppresses the differentiation and proliferation of GMP, possibly via the inhibition of the transcription factors Runx1 and C/EBPα.

An interesting unexpected finding of this study is that Twist-2 promotes the production of the important regulatory cytokine IL-10 [65] by differentiated myeloid cells. The etiology of the lethal inflammatory phenotype of Twist-2 KO mice is distinct, but not well understood [35]. The enhanced production of proinflammatory cytokines in combination with the reduced production of anti-inflammatory cytokine IL-10 may contribute to the lethal inflammation of Twist-2 KO mice [35,36]. In addition, hyperproliferation of myeloid progenitors and the expanded population of differentiated inflammatory myeloid cells found in Twist-2 KO mice may perpetuate the lethal inflammation. Different from the constitutive expression of Twist-2 in GMP, the expression of Twist-2 in differentiated myeloid cells such as DCs and macrophages was induced in response to TLR agonists such as LPS. Twist-2 was previously shown to directly bind the p65 subunit of NF-κB and inhibit the transactivation and production of inflammatory cytokines such as TNFα, IL-1, and IL-6 [35]. In this study, we found that the production of Th1-polarizing cytokines such as IL-12 and IFNγ was also critically regulated by Twist-2. This is in agreement with a recently study describing a role for Twist-1 in regulating Th1 cytokines in T-cells [66]. We further demonstrated that the mechanism for the suppression of IL-12 production by Twist-2 involves inhibition of NF-κB activity, not via the direct binding of Twist-2 to E-boxes in the IL-12 promoter (unpublished data). It was previously reported that C/EBPα and NF-κB p50 or p65 directly interact and cooperate to activate proinflammatory genes [67]. Our finding of the inhibition of C/EBPα by Twist-2 raises an interesting possibility that the critical regulation of proinflammatory cytokines by Twist-2 may be a result of the inhibition of both NF-κB and C/EBPα proteins by Twist-2. Moreover, Twist-2 was found to have a critical role in maintaining endotoxin tolerance. Thus, Twist-2, in addition to its critical role in regulation of myeloid lineage differentiation and the proliferation of myeloid progenitors, uniquely regulates proinflammatory responses to prevent excessive inflammation by inhibiting proinflammatory cytokines and promoting anti-inflammatory cytokine production.

IL-10 is a potent anti-inflammatory cytokine and plays a critical role in maintaining homeostasis of the immune system and protecting the host from excessive inflammation [65]. Despite its biological importance, the regulation of IL-10 gene expression remains poorly defined. In this study, we found that Twist-2 promotes the expression of IL-10, not via direct activation of the IL-10 promoter, but possibly through the activation of c-Maf transcription. We demonstrated that Twist-2 is likely a transcriptional activator of c-Maf. bHLH transcription factors have been known to function as activators or repressors, depending on the partners or cofactors [45,48]. c-Maf is the cellular counterpart of oncogenic v-Maf in the acute transforming avian retrovirus AS42 that induces musculoaponeurotic fibrosarcoma (Maf) [68]. c-Maf is a member of the family of basic region–leucine zipper domain transcription factors, and the target genes of c-Maf in various cell lineages include the insulin gene in islet β cells and IL-4 and IL-10 in T cells and myeloid cells [49,69,70]. Collectively, the data suggest a possible positive role of Twist-2 in the Maf transcription and subsequent IL-10 production in myeloid cells. Further studies are warranted to investigate the role of Twist-2 in regulation of innate and adaptive immunity.

## Materials and Methods

### Mice.

Twist-2 heterozygous null mice were crossed to generate Twist-2 KO mice [35]. In all experiments, WT littermates were used for controls. Mice were maintained in a pathogen-free barrier facility, and all experiments were performed in accordance with the Baylor College of Medicine Institutional Animal Care and Use committee.

### Immunostaining and cell sorting.

Single-cell suspensions were prepared from indicated solid organs of mice 10–12 d old. Red blood cells were lysed with Tris-ammonium chloride lysis buffer. Peripheral blood mononuclear cells (PBMC) were isolated from peripheral blood by gradient centrifugation. Fc receptor block was performed using anti-mouse CD16/CD32 Fc block antibody (BD Pharmingen), except in cases when the Fc receptor was used to identify CMP or GMP. Cells were stained with combinations of fluorescein isothiocyanate, phycoerythrin, allophycocyanin, peridinin chlorophyll protein, or phycoerythrin-Cy7–conjugated CD4, CD8, IgM, IgD, CD11b, Gr-1, Annexin-V, lineage cocktail (CD4, CD8, Ter-119, CD11b, Gr-1, and B220), IL-7R, Sca1, c-Kit anti-mouse antibodies (BD), or APC-conjugated F4/80 (Caltag Labs), or biotinylated FcγRIII/II followed by staining with streptavidin-PerCP Ab (BD). Cells were analyzed and sorted on a FACSaria (BD) as described [71]. FACS data were analyzed using FlowJo Software (TreeStar).

### Hematopoietic colony assays.

Single-cell suspensions of BM after ammonium chloride lysis were plated into 60-mm dishes (2 × 10^4^ cells/plate) with MethoCult M3001 (containing only GM-CSF) or GF M3434 as indicated (StemCell Technologies). Cells were cultured at 37 °C, 5% CO_2_, and hematopoietic colonies were counted and scored after 12 d of incubation, or as indicated.

### CBC analysis and histology.

Peripheral blood from Twist-2 KO mice and WT littermates 10–12 d old was collected via retro-orbital bleed into Microvettes coated with EDTA (SARSTEDT). Automated complete blood count with differential was performed using an Advia 120 (Bayer Diagnostics, SIEMENS). Peripheral blood smears were generated and imaged (BX51 Olympus Microscope). For whole bone mounts, femurs were fixed and processed for paraffin embedding and stained with hematoxylin and eosin (H&E). For BM histology, femurs were incised lengthwise, and BM was gently touched to the surface of a glass slide, followed by Wright-Giemsa staining and pathological analysis.

### ELISA.

BM-derived DCs were generated as previously described [71,72]. Primary CD11c^+^ splenic DCs were purified from single-cell suspensions of splenocytes using CD11c^+^ magnetic bead columns (Miltenyi Biotec). DCs were plated (1 × 10^6^ cells/ml) into 24- or 96-well plates and stimulated with 100 ng/ml LPS for 24 h. For some assays, WT and KO BMDCs were transfected with Twist-2 expression vectors using the AMAXA Mouse Dendritic cell Nucleofector kits [73]. Briefly, 2.5 × 10^5^ BMDC per condition were electroporated with either 2 μg of pcDNA3.1 empty vector control or 2 μg of Twist-2 expression construct and plated into 48-well plates in RPMI supplemented with 10% fetal bovine serum (FBS), 100 U/ml penicillin, 100 μg/ml streptomycin, 2 mM l-glutamine, and 20 ng/ml GM-CSF. Cells were rested overnight and then stimulated with 100 ng/ml LPS for 24 h. Supernatants were harvested and analyzed by ELISA (BD).

### Luciferase reporter assay.

COS7 cells (0.6 × 10^6^ cells/well) or 293T cells were seeded into 6-well plates and cultured for 24 h in DMEM supplemented with 10% fetal bovine serum (FBS), 100 U/ml penicillin, 100 μg/ml streptomycin, and 2 mM l-glutamine. Cells were washed with serum-free (SF) DMEM and cotransfected with indicated plasmid expression vectors using Geneporter (Gene Therapy Systems). The Runx1, C/EBPα, and PU.1 expression constructs and respective luciferase reporter constructs have been described [74,75]. pcDNA3.1 empty control vector was added so that each well received the same total amount of DNA. After 4 h, an equal volume of DMEM supplemented with 20% FCS was added to the wells. Cells were cultured for an additional 24 h, and luciferase activity of cell extracts was measured on a luminometer normalized to total protein concentrations using the Dual-Luciferase Reporter Assay System (Promega).

### EMSA.

Sorted GMP (2 × 10^5^ cells) from Twist-2 KO mice or WT littermates were lysed in 25 μl of NTN lysis buffer containing phosphatase inhibitor cocktail 1 and 2 (SIGMA) and Complete Mini Protease Inhibitor Cocktail (Roche). Lysates were incubated on ice for 45 min and cleared by centrifugation at 14,000×*g* for 10 min. Cleared lysates were reacted for 20 min at room temperature (RT) with biotinylated oligo duplexes (Operon) containing the wild-type or mutated RUNX1 consensus binding site from the M-CSF promoter: WT sense: 5′-AGCTAACTCTGTGGTTGCCTTGCCT-3′; WT antisense: 5′-TCGAAGGCAAGGCAACCACAGAGTT-3′; mutant sense: 5′-AGCTAACTCTGT*ACA*TGCCTTGCCT-3′; mutant antisense: 5′-TCGAAGGCAAGGCATGTACAGAGTT-3′. Nonbiotinylated oligos were used as additional controls. Binding reactions and EMSA were set up and performed using the Lightshift Chemiluminescent EMSA Kit (Pierce) and Chemiluminescent Nucleic Acid Detection Module (Pierce) according to the manufacturers' protocols. Briefly, reacted samples were immediately loaded and electrophoresed on 6% DNA retardation gels (Invitrogen) in 0.5× TBE and transferred to BrightStar-Plus positively charged nylon membrane (Ambion). DNA was crosslinked at 120 mJ/cm^2^ using a GS Gene Linker UV Chamber (Bio-Rad). Membranes were blocked and biotin-labeled DNA was detected and visualized using streptavidin-HRP followed by ECL reagent (Pierce).

### RT-PCR.

For real-time quantitative RT-PCR, total RNA was isolated from indicated cell type using the RNeasy kit (Qiagen). First-strand cDNA was synthesized from 200 ng of total RNA for hematopoietic stem or progenitor cells or from 4 μg of total RNA for mature cells using the Superscript II First-Strand Synthesis System (Invitrogen). Real-time quantitative RT-PCR was set up using TaqMan Universal PCR Master Mix (Applied Biosystems) and analyzed on an ABI Prism 7900HT Sequence Detection System (Applied Biosystems). TaqMan gene expression assays for indicated genes were ordered from Applied Biosystems. All data were normalized to 18S rRNA internal controls [72,76]. For semiquantitative RT-PCR 25 ng of first-strand cDNA was amplified using Twist-1– or Twist-2–specific primers (sequences available upon request) for 40 cycles using HPRT primers as controls.

### ChIP and immunoprecipitation.

3T3 fibroblasts were grown in 10-cm plates until semiconfluent. Cells were fixed in 1% paraformaldehyde, and ChIP was performed using the EZ ChIP Kit according to the manufacturer's instructions (Upstate). Briefly, cell lysate from approximately 20 × 10^6^ cells was sonicated using a Bath sonicator (Bioruptor). Precleared lysates were incubated with Anti-RNA Polymerase II Ab (Clone CTD4H8), normal mouse IgG, or anti-Twist-2 antibodies (H-81 sc-15393) overnight at 4 °C. Immunocomplexes were collected, and purified DNA was analyzed by RT-PCR using indicated primers. Densitometry was performed to quantify relative band intensity as normalized to input. Primer sequences would be provided upon request. For immunoprecipitation, COS7 cells in 10-cm plates were transfected with indicated plasmids and cultured for 48 h. Cells were lysed in 500 μl of NTN NP-40 lysis buffer containing protease inhibitor cocktail (Roche) and lysate was homogenized by passing though a 21G needle. Lysate was incubated with antibodies overnight at 4 °C followed by addition of Protein G-Sepharose beads (Zymed) and incubation for 2 h. Beads were washed four times, resuspended in 70 μl of SDS loading buffer, and then incubated at 95 °C for 8 min. Samples were analyzed by western blotting with either anti-FLAG or anti-Myc primary antibodies (Sigma) and secondary antibody conjugates (Sigma).

### Statistics.

For statistical analysis, we performed the Student *t*-test, and *p*-values of less than 0.05 were considered statistically significant. Results are presented as means plus or minus standard errors (SE).

## Supporting Information

Figure S1 Twist-2 Expression in CMP and GMP mRNA expression of known myeloid transcription factors and bHLH factors in CMP and GMP from GEO dataset GSE3722 (http://www.ncbi.nlm.nih.gov/geo/query/acc.cgi?acc=GSE3722). Flow sorting scheme for hematopoietic stem and progenitor cells.
(205 KB PPT)

Figure S2 Electrophoretic Mobility Shift Assay of Sorted GMPs Whole-cell lysates of sorted Lin^−^IL7R^−^Sca-1^−^c-Kit^+^CD34^+^FcγRII/III^high^ GMPs (2 × 10^5^) from WT and Twist-2 KO mice were reacted with biotinylated oligos containing the endogenous Runx1 consensus binding site (Runx1 probe) or mutated Runx1 consensus binding site (mutant Runx1 Probe), and EMSA was performed. Arrow indicates specific Runx1 band retardation. An asterisk (*) indicates nonspecific band. Densitometry was performed on WT and KO bands indicated with arrow and was normalized to background in each lane. Experiment was repeated twice with similar results.
(81 KB PPT)

Figure S3 Flow Cytometric Analysis of Surface Markers on BMDCs With or Without LPS Stimulation for 24 h (716 KB PPT)

Figure S4 Images and Flow Cytometric Analysis of BMDC Using the AMAXA Mouse Dendritic Cell Nucleofector Kits [73] Flow cytometric analysis was performed to quantify transfection efficiency and viability with PI staining of DCs transfected with a green fluorescent protein (GFP) expressor.
(552 KB PPT)

## Abbreviations

bHLH: basic helix-loop-helix
BM: bone marrow
BMCP: basophil mast cell progenitor
BMDC: bone marrow-derived dendritic cell
BrdU: bromodeoxyuridine
ChIP: chromatin immunoprecipitation
CMP: common myeloid progenitor
DC: dendritic cell
EMSA: electrophoretic mobility shift assay
GM-CSF: granulocyte-macrophage colony-stimulating factor
GMP: granulocyte macrophage progenitor
HLH: helix-loop-helix
HSC: hematopoietic stem cell
IL: interleukin
KO: knockout
NF-κB: necrosis factor κB
PI: propidium iodide
RT-PCR: reverse transcription PCR
SCL: stem cell leukemia factor
TNFα: tumor necrosis factor-α
WT: wild-type
